# Autophagy attenuates osteoarthritis in mice by inhibiting chondrocyte pyroptosis and improving subchondral bone remodeling

**DOI:** 10.17305/bjbms.2022.7677

**Published:** 2023-01-06

**Authors:** Jiangbo Yan, Gangning Feng, Yong Yang, Dong Ding, Long Ma, Xin Zhao, Xiaolei Chen, Hui Wang, Zhirong Chen, Qunhua Jin

**Affiliations:** 1 Clinical College, Ningxia Medical University, Yinchuan, China; 2 Orthopedics Ward 3, The General Hospital of Ningxia Medical University, Ningxia Medical University, Yinchuan, China; 3 Hand and Ankle Department, Shandong Provincial Hospital Affiliated to Shandong First Medical University, Jinan, China

**Keywords:** Osteoarthritis (OA), chondrocyte, autophagy, pyroptosis, subchondral bone

## Abstract

Osteoarthritis (OA) is an age-related degenerative disease characterized by cartilage degeneration and abnormal bone remodeling in the subchondral bone. Autophagy maintains cellular homeostasis by self-phagocytosis. However, the underlying mechanisms of autophagy on the pathological progression of OA are still unknown. This study assessed the effects of autophagy on cartilage and subchondral bone in a mouse OA model. A mouse OA model was induced using destabilization of the medial meniscus surgery. Assessment was performed by histomorphology, microcomputed tomography, immunohistochemical, immunofluorescent, and tartrate-resistant acid phosphatase staining. Our data revealed that autophagy can significantly delay the pathological progression of OA by increasing the thickness of hyaline cartilage and decreasing the thickness of calcified cartilage, increasing the subchondral bone volume fraction and bone mineralization density, and decreasing trabecular separation in the early stages of OA (two weeks), whereas the opposite is true in the late stages of OA (eight weeks). Mechanistically, activation of autophagy in cartilage increased the expression of type II collagen, decreased the expression of matrix metalloproteinase 13, and decreased the pyroptosis mediated by NOD-like receptor protein 3 inflammasome by decreasing the expression of NOD-like receptor protein 3 inflammasome, caspase-1, gasdermin D, and interleukin-1β. In the subchondral bone, activation of autophagy decreased the generation of mature osteoclasts at the early stages of OA (two weeks) mainly by reducing the receptor activator for nuclear factor-κB ligand/osteoprotegerin ratio, while it decreased osteoblastogenesis by reducing Runt-related transcription factor 2 expression significantly in the late stages of OA (eight weeks). In conclusion, autophagy may delay the pathological progression of OA in mice by inhibiting chondrocyte pyroptosis and improving subchondral bone remodeling.

## Introduction

Knee osteoarthritis (OA) is one of the most common skeletal disorders characterized by loss of articular cartilage, synovitis, fibrosis, and inflammation of the infrapatellar fat pad, subchondral sclerosis, and osteophyte formation [1, 2]. At present, the etiology and pathogenesis of OA have not been fully clarified and are generally considered to result from a combination of biological and mechanical factors [3]. Pathological death of chondrocytes, the important component of cartilage of the tibial plateau and femoral condyles in knee, is a major biological factor in OA [4]. Thus, inhibiton of chondrocyte death is of extraordinary significance for delaying the pathological process of OA.

Pyroptosis is a form of cell death discovered in recent years in which DNA damage, chromatin condensation, cell swelling, and numerous irregular protrusions on the cell membrane surface occur before the cell membrane ruptures [5]. The canonical activation pathway of pyroptosis is mediated by the NOD-like receptor protein 3 (NLRP3) inflammasome, which requires the pattern recognition receptor (PRR) as a sensor, in most cases the adaptor apoptosis-associated specific protein containing a C-terminal caspase recruitment domain (ASC) and the cysteine protease caspase-1 [6]. In the conventional activation pathway, extracellular stimulatory signals are transmitted intracellularly causing NLRP3 inflammasome initiation and activation [7]. The priming step upregulates the expression of inflammasome [8]. Subsequently, signals from the upstream cause NLRP3 to form a complex with ASC and caspase-1 – the NLRP3 inflammasome, which in turn cleaves gasdermin D (GSDMD) to form GSDMD-N mosaic on the cell membrane that induces the development of pyroptosis. This complex can also promote the activation of interleukin-1β (IL-1β) and IL-18, which flow through the pores formed above into the extracellular space and mediate inflammatory processes [9]. There is evidence that the NLRP3 inflammasome participates in the pathogenesis of OA. The underlying mechanism may be the activation of toll-like receptors and nuclear factor kappa B (NF-κB) signaling pathways that lead to the pathological changes in OA, such as cartilage degeneration and synovial inflammation [10]. Currently, the role and underlying mechanisms of NLRP3-mediated pyroptosis in OA remain unknown.

Autophagy is a process of self-phagocytosis and self-renewal, which maintains the homeostasis of the intracellular environment through the degradation and functional recovery of dysfunctional proteins and organelles [11]. It has been shown that the activation of autophagy by rapamycin (RAPA) can delay the progression of OA through multiple pathways, including the regulation of reactive oxygen species (ROS), the expression of matrix metalloproteinase 3 (MMP-3), MMP-13, and tumor necrosis factor-α (TNF-α), [12–14]. A recent study showed that activation of autophagy can prevent the occurrence of pyroptosis [15]. At the same time, the inhibition of autophagy can exacerbate pyroptosis [16]. Accordingly, we hypothesized that a key step in the regulation of pyroptosis by autophagy may be the degradation of the NLRP3 inflammasome. This raises the question of whether activation of autophagy by RAPA delays the progression of OA associated with chondrocyte pyroptosis in a mouse OA model.

Mechanical factors are another important factor affecting the development of OA [17]. Some researchers believe that abnormal bone remodeling in the subchondral bone may be the initiating factor in the pathogenesis of OA [18]. Abnormal mechanical stress can disrupt joint homeostasis, as evidenced by subchondral bone remodeling and osteophyte formation [19]. Bone resorption resulting from active osteoclasts in the subchondral bone is predominant in the early stages of OA, which is characterized by reduced bone volume and can present as stress microfractures. In the later stages, bone formation resulting from enhanced osteoblast activity in the subchondral bone is predominant, which is characterized by increased bone volume and bone mineral density, with significant subchondral osteosclerosis [20]. It has been shown that autophagy-dependent mitochondrial function plays an important role in osteoclast differentiation and maturation [21]. Autophagy in osteoblasts is involved in mineralization processes and bone homeostasis [22]. However, it is unclear whether subchondral bone autophagy affects the progression of OA by regulating the balance between subchondral bone resorption and bone formation. Overall, based on these data, we hypothesized that autophagy affects the progression of OA by inhibiting chondrocytes pyroptosis and subchondral bone remodeling. To address this hypothesis, this study constructed an OA model in mice was with destabilization of the medial meniscus (DMM) to illustrate the role of autophagy in chondrocyte pyroptosis and subchondral bone remodeling in OA. Through the study, we revealed the underlying mechanism by which autophagy affects the progression of OA pathology.

## Materials and methods

### Animals and DMM-induced OA models

All animals in this study were healthy, wild-type, adult male C57BL/6 mice (specific-pathogen-free [SPF] grade), eight weeks old, weighing 20–25 g, obtained from the Laboratory Animal Center of Ningxia Medical University, Yinchuan, China. All experimental mice were fed in the SPF environment, maintaining a temperature of 22 ± 1 ^∘^C and a humidity of 55% in a 12-h light/dark cycle, and were allowed free access to food and water. The *in vivo* experiments were in accordance with the ARRIVE guidelines [23]. In this experiment, surgically induced OA was caused by DMM. Briefly, mice were anesthetized by intraperitoneal injection of 1% pentobarbital sodium in PBS (60 mg/kg), the right knee joint capsule was incised, and the meniscus tibial ligament was transected using micro-scissors so that the anterior horn of the medial meniscus was freed. The sham operation was performed after opening the right knee joint capsule, without treating of the meniscus tibial ligament, and direct suturing was performed after a normal examination.

### Experimental design and processing

**Figure 1.**
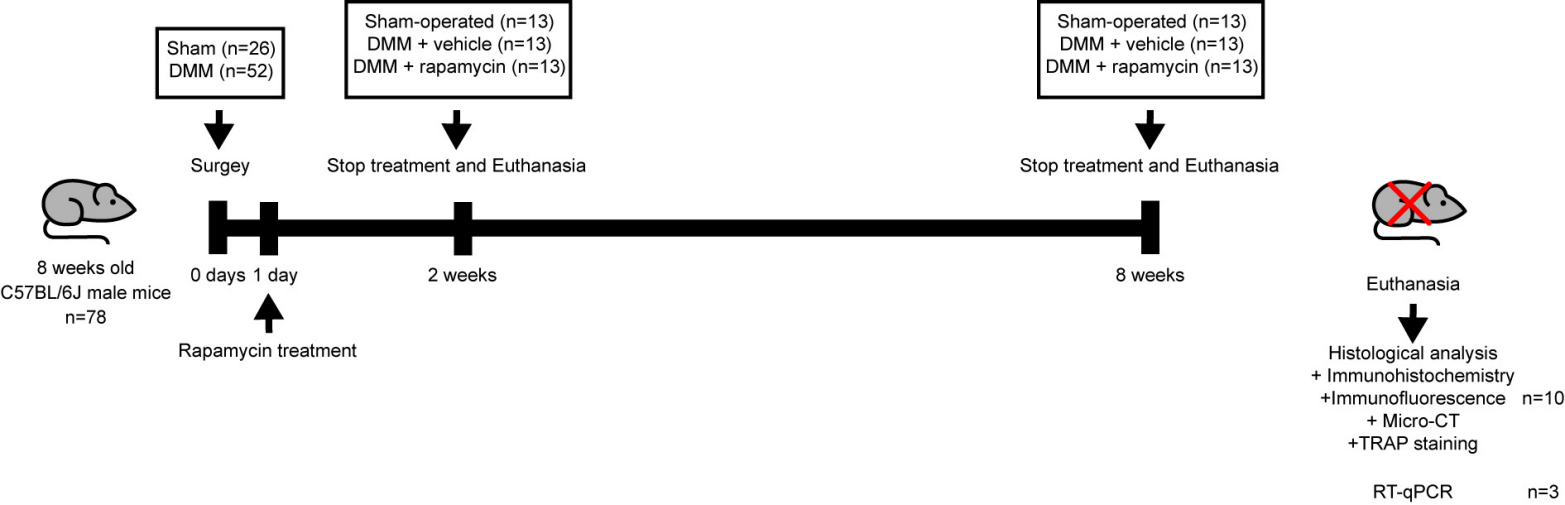
**Schematic diagram of animal experiment**. DMM: Destabilization of the medial meniscus; micro-CT: Microcomputed tomography; TRAP: Tartrate-resistant acid phosphatase; RT-qPCR: Quantitative real-time PCR.

A total of 78 mice were randomly and equally assigned to 3 groups (with 26 mice in each group), including the control group (sham group), the OA group (model group), and the rapamycin (Cat. no. S1039; Selleck Chemicals Co., Ltd., Houston, TX, USA) treatment group (RAPA group). As described in our previous study [24], RAPA was dissolved and stored in dimethyl sulfoxide (DMSO) at the concentration of 25 mg/mL. The Bulk solution was diluted in phosphate-buffered saline (PBS) for intraperitoneal injection and administered daily at a dose of 1 mg/mL body weight until sacrifice (two or eight weeks after surgery). The sham and model groups were injected with normal saline or DMSO as a control, respectively. The experimental design of the animal studies is shown in Figure 1.

### Histological evaluation of articular cartilage degeneration

Right knees from 10 mice per group were collected for subsequent experiments, which were fixed in 4% paraformaldehyde (PFA) for 24 h and subsequently rinsed hourly with PBS for 6 h, after which they were decalcified with 10% ethylenediaminetetraacetic acid (EDTA) decalcifying solution (AR1071; Wuhan Boster Biological Technology, Wuhan, China) for 2 weeks. Knees were dehydrated with graded ethanol and embedded in paraffin. Knees were sectioned coronally using a thickness of 4 µm, which was stained with a hematoxylin and eosin (HE) kit (G1053; Servicebio, Wuhan, China) to determine the thickness of hyaline cartilage (HC) and calcified cartilage (CC), as previously described [25]. Briefly, sections were deparaffinized twice in xylene for 20 min each, then processed twice in absolute ethanol for 10 min each and then in 75% alcohol for 5 min each. Sections were stained with HE for 5 min and then dehydrated in 75% and 95% alcohol for 5 min. For safranin O-fast green staining (G1005; Servicebio, Wuhan, China), sections were deparaffinized as described above and then stained with Fast Green for 5 min, washed, dehydrated, and counterstained with Safranin-O for 5 min. Mouse cartilage degeneration was histologically scored by double-blind observation using a modified Osteoarthritis Research Society International (OARSI) scoring system [26]. Five random views from three sections per mouse were visualized by the Olympus DP71 light microscope.

**Table 1:** Primer sequence used in reverse transcription-quantitative PCR

**Gene**	**F-Primer**	**R-Primer**
*NLRP3*	5’-TGCCGTGGTCTCTTCTCAAG-3’	5’-GTCGAAGCAGCATTGATGGG-3’
caspase-1	5’-TGATGGCATTAAGAAGGCCCA-3’	5’-TCCAAGTCACAAGACCAGGC-3’
*GSDMD*	5’-ATGGGAACATTCAGGGCAGAG-3’	5’-ACCTCAGTGATCTGCACTTCC-3’
IL-1β	5’-TGACGGACCCCAAAAGATGAAG-3’	5’-AGCTCTTGTTGATGTGCTGC-3’
β-actin	5’-GTGCTATGTTGCTCTAGACTTCG-3’	5’-ATGCCACAGGATTCCATACC-3’

NLRP3: NOD-like receptor protein 3; GSDMD: Gasdermin D protein; IL-1β: Interleukin-1β.

### Immunohistochemistry and immunofluorescence

Paraffin-embedded sagittal sections of mouse knees were sliced 4-µm thick, deparaffinized with xylene and dehydrated with graded alcohols. The slides were incubated with 3% hydrogen peroxide for 15 min and then blocked with 5% goat serum for 30 min at 37 ^∘^C and incubated 10 h at 4 ^∘^C with the following primary antibodies: anti-LC 3 (1:100, 14600-1-AP; ProteinTech), anti-MMP-13 (1:300, ab39012; Abcam), anti-Col II (1:300, ab34712; Abcam), anti-NLRP3 (1:200, ab214185; Abcam), anti-caspase-1 (1:200, 22915-1-AP; ProteinTech), anti-GSDMD (1:200, ab219800; Abcam), anti-IL-1β (1:300, ab205924; Abcam), anti-RANKL (1:200, ab9957; Abcam), anti-OPG (1:200, ab73400; Abcam), and anti-Runx2 (1:200, ab192256; Abcam). For immunohistochemical (IHC) staining, sections were incubated with a secondary antibody for 1 h at 20 ^∘^C. The color was then developed using 3,3’-diaminobenzidine (DAB) (ZLI-9018; Zhongshan Jinqiao Biotechnology Co.), followed by counterstaining with hematoxylin. For immunofluorescence (IF) staining, sections were incubated using secondary antibody conjugated with fluorescence (1:500, ab150077; Abcam) for 1 h at 37 ^∘^C in a dark environment. Five random views from three sections per mouse were visualized by fluorescence microscopy.

### Total RNA extraction and reverse transcription-quantitative polymerase chain reaction

Total RNA was extracted from three knees in each group following the operating instructions of the total RNA extraction kit (AP-MN-MS-RNA-250; Axygen, Union City, CA, USA). Complementary DNA (cDNA) synthesis was performed following the TransScript All-in-One First-Strand cDNA Synthesis kit (AT341-01; TransGen Biotech, Beijing, China) operating instructions to synthesize cDNA using 1 µg of total RNA. Quantitative real-time PCR experiments (RT-qPCR) was subsequently performed to quantify the expression of relevant genes. The RT-qPCR reaction conditions consisted of 45 cycles of 94 ^∘^C for 30 s, 54 ^∘^C for 30 s, and 72 ^∘^C for 34 s. The primer sequences are listed in Table 1.

β-actin was used as the quantitative internal control gene. The 2^-ΔΔCq^ method was used to calculate the relative gene expression [27].

### Microcomputed tomography

A microcomputed tomography (micro-CT) device (Bruker, Billerica, MA, USA) was used to scan the mouse knee joint. Data analysis software (CTAnv1.9; Bruker Belgium S.A./N.V.) and three-dimensional (3D) model visualization software (CTVol v2.0; Bruker Belgium S.A./N.V.) were used to analyze the data. We determined the quantitative morphological metrics based on the 3D morphology-based microtomographic data [28]. The area we measured was between the tibial growth plate and the tibial plateau. Evaluation indicators included the bone volume fraction (bone volume/trabecular volume [BV/TV]; %), bone mineral density (BMD; g/cm^3^), and trabecular separation (Tb.Sp; mm).

**Figure 2.**
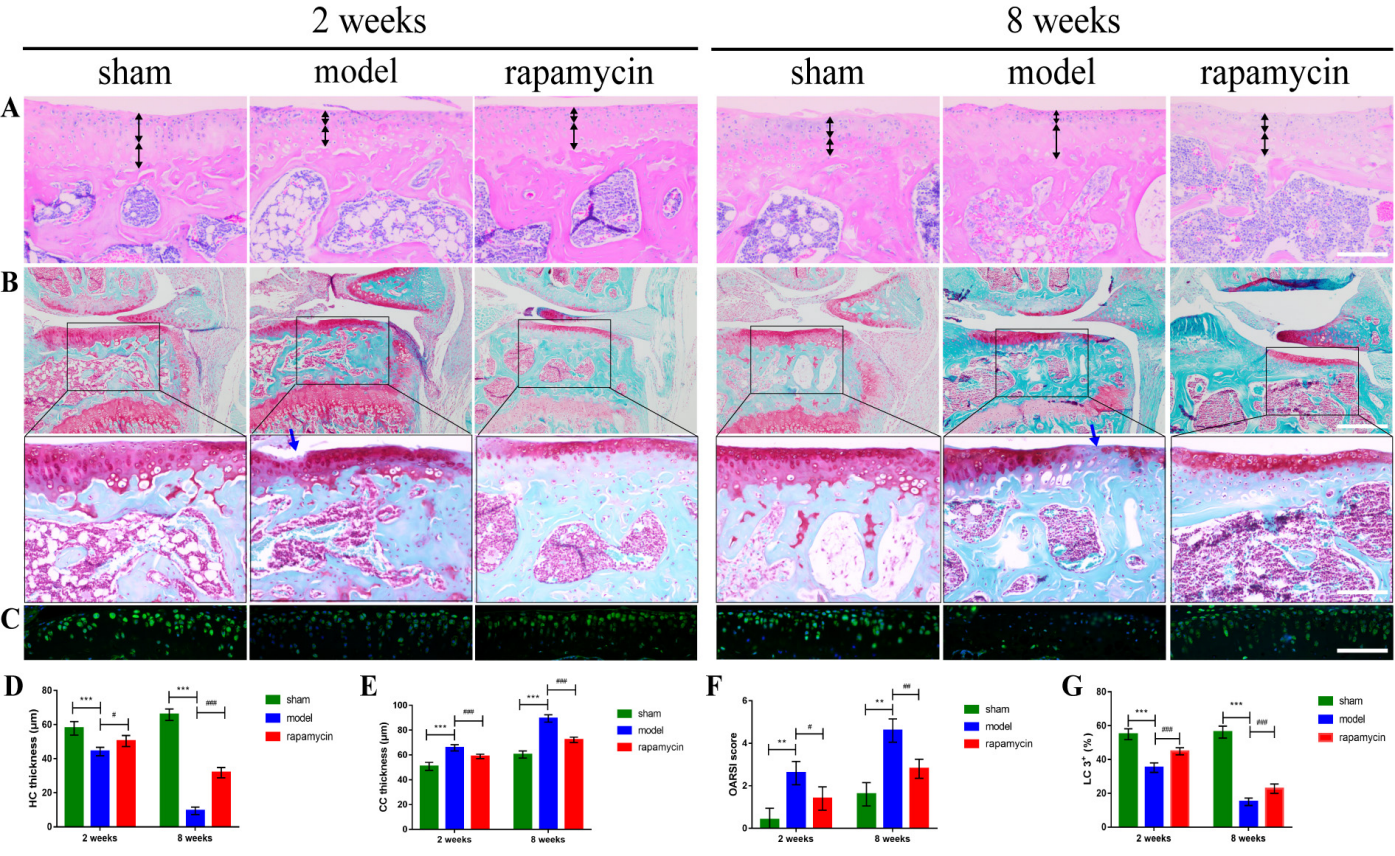
**Effect of autophagy on cartilage degradation in a mouse OA model**. Representative pictures of HE staining (A) (scale bar: 100 µm), safranin O-fast green staining (B) (scale bar: 50 µm and 100 µm), and IF staining (C) (scale bar: 100 µm) of knee joints at two and eight weeks after DMM surgery in mice. Black bidirectional arrows indicate the thickness of HC and CC, and blue arrows indicate cartilage degeneration. The quantitative analysis of HC and CC thickness (D, E), OARSI score (F), and LC3 expression (G) in tibial plateau cartilage. *n* = 5 per group, all data presented as means ± standard deviations. **P < 0.01; ***P < 0.001; ^#^*P* < 0.05; ^##^*P* < 0.01; ^###^*P* < 0.001. OA: Osteoarthritis; DMM: Destabilization of the medial meniscus; IF: Immunofluorescence; HC: Hyaline cartilage; CC: Calcified cartilage; OARSI: Osteoarthritis Research Society International; LC3: Light chain 3; HE: Hematoxylin and eosin.

### Tartrate-resistant acid phosphatase staining

Paraffin-embedded sagittal sections of mouse knees were sliced to 0.4-µm thick, deparaffinized with xylene, and dehydrated with graded ethanol for subsequent experiments. Following the instructions for use of the tartrate-resistant acid phosphatase (TRAP) staining kit (G1005; Servicebio, Wuhan, China), the prepared working solution was placed on the tissue section at 37 for incubation for 2 h, washed 3 times with distilled water, and then hematoxylin was used for staining for 5 min. The number of TRAP^+^ osteoclasts in five random views from three sections per mouse was detected by the Olympus DP71 light microscope.

### Ethical statement

The relevant study protocol and contents were approved by the Animal Experiment Ethics Committee of Ningxia Medical University (Yinchuan, China; protocol no. 2020-115).

### Statistical analysis

GraphPad Prism 7.0 (GraphPad software, San Diego, CA, USA) was used for statistical analysis. All data were presented as mean ± standard deviation (SD). After testing the data for homogeneity of variance, one-way analysis of variance (ANOVA) and Tukey’s multiple comparison test were used to compare the data. Nonparametric data (OARSI scores) were analyzed using the Kruskal–Wallis H test followed by Dunn’s test. A *P*-value <0.05 was considered statistically significant.

## Results

### Effect of autophagy on the pathological progression of OA cartilage

To investigate the effect of autophagy on disease progression in a DMM-induced OA model, we first performed safranin O-fast green staining on mouse knee cartilage collected two and eight weeks after DMM surgery. The results showed that the cartilage surface of the tibial plateau of the knee joint in the sham group was continuous and smooth, and the tidal line was clearly visible. Compared with the sham group, the model group showed destruction of the cartilage surface, discontinuity, and massive proteoglycan loss and even loss of hyaline cartilage (HC) and exposure of calcified cartilage (CC) eight weeks after DMM surgery. A significant increase in articular cartilage thickness as well as alleviation of cartilage damage was observed in the RAPA treatment group compared with the model group (Figure 2A and 2B). The OARSI score showed that the score of the model group was significantly higher than that of the sham group, and the OARSI score of the RAPA treatment group was significantly lower than that of the model group (Figure 2F). At the same time, the thickness of HC was significantly reduced, and the thickness of CC was significantly increased in the model group compared with the sham group, whereas abnormally thinned HC and abnormally thickened CC were significantly improved in the RAPA group compared with the model group (Figure 2D and 2E). The IF staining results showed that the expression of light chain 3 (LC3) in chondrocytes was significantly decreased in the model group compared with the sham group, and the expression of LC3 was significantly increased in the RAPA treatment group compared with the model group (Figure 2C and 2G).

### Effect of autophagy on the metabolism of OA chondrocytes

**Figure 3.**
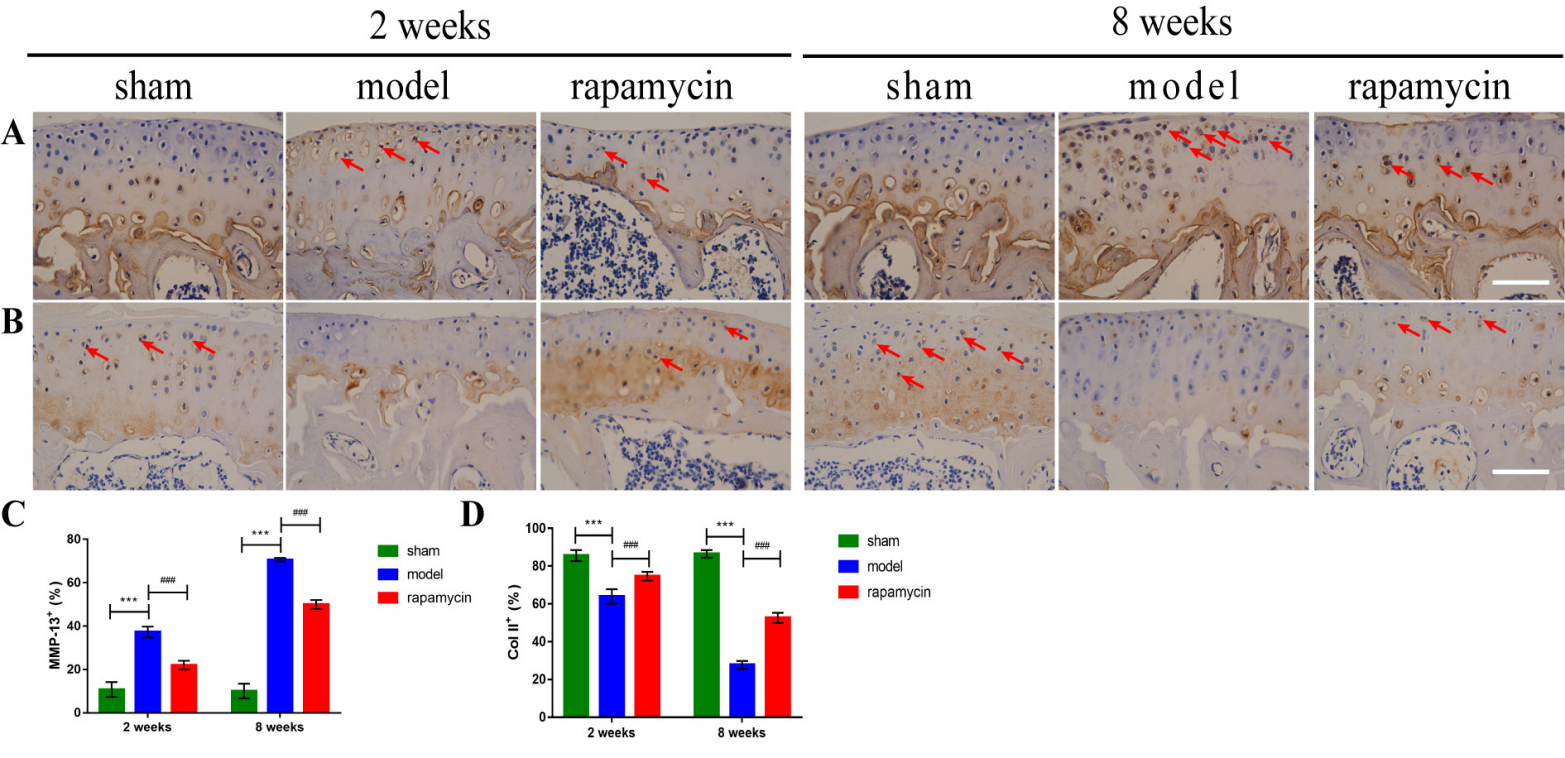
**Effect of autophagy on chondrocyte metabolism in a mouse OA model.** Representative pictures of IHC staining for MMP-13 (A) and Col II (B) in chondrocytes of knee tibial plateau at two and eight weeks after DMM surgery in mice, and quantitative analysis of MMP-13 (C) and Col II (D) expression. Positive cells were shown as red arrows. Scale bar: 100 µm; *n* = 5 per group, all data presented as means ± standard deviations. ***P < 0.001; ^###^*P* < 0.001. OA: Osteoarthritis; IHC: Immunohistochemistry; DMM: Destabilization of the medial meniscus; MMP-13: Matrix metalloproteinase 13; Coll II: Type II collagen.

To investigate the effect of autophagy on chondrocyte metabolism in a DMM-induced OA model, we performed IHC staining for MMP-13 and type II collagen (Col II) in mouse knee cartilage collected two and eight weeks after DMM surgery. This showed that the number of MMP-13-positive cells in the DMM was significantly increased compared with the sham group, and the abnormally expressed MMP-13 was significantly reduced in the RAPA treatment group compared with the model group (Figure 3A and 3C). At the same time, the number of Col II-positive cells in the model group was significantly lower compared with the sham group, and abnormally expressed Col II was significantly increased in the RAPA treatment group compared with the model group (Figure 3B and 3D).

### Effect of autophagy on chondrocyte pyroptosis in OA

**Figure 4.**
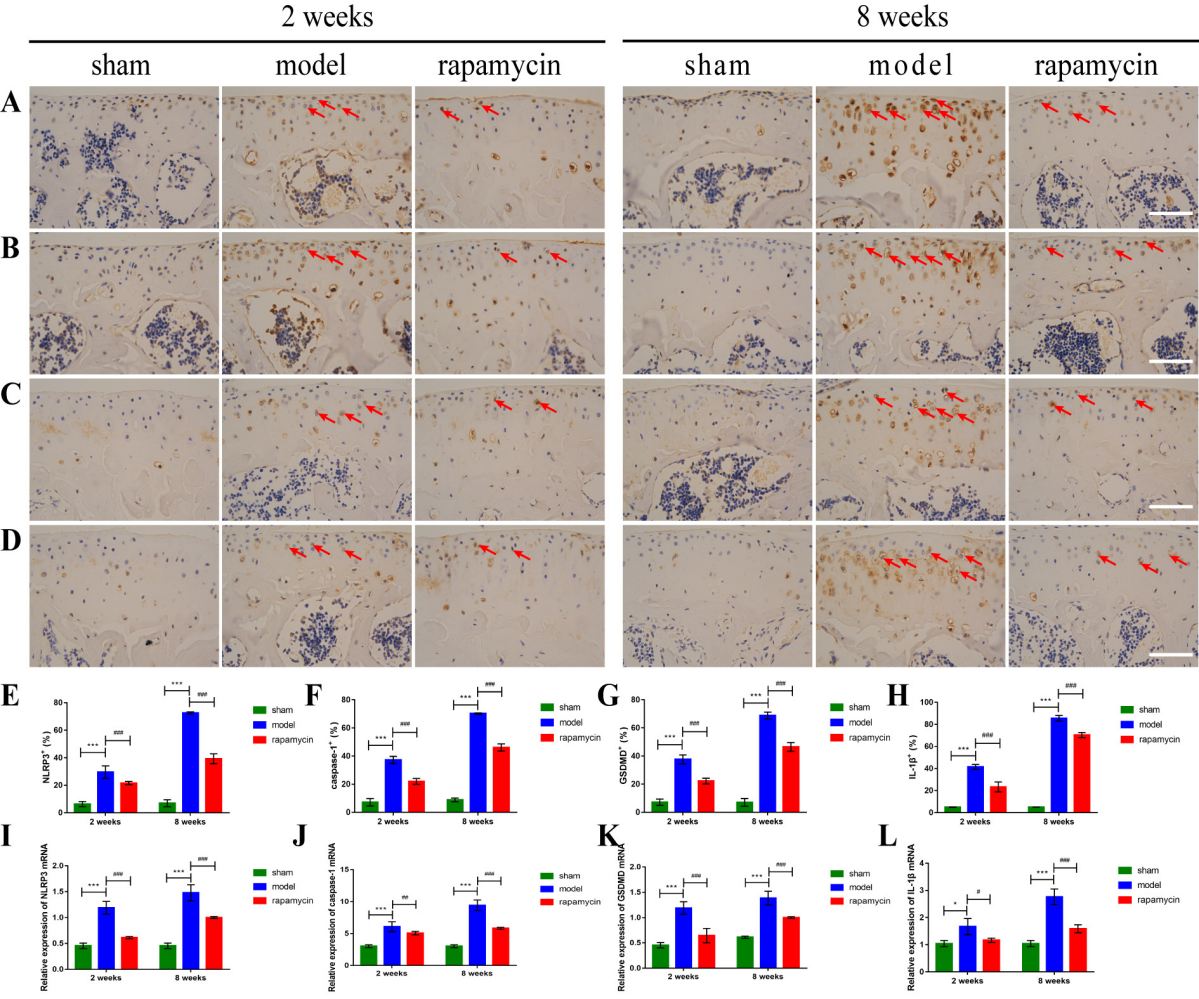
**Effect of autophagy on chondrocyte pyroptosis in a mouse OA model.** Representative images of IHC staining of NLRP3 (A), caspase-1 (B), GSDMD (C), IL-1β (D) in chondrocytes of the knee tibial plateau at two and eight weeks after DMM surgery in mice, and quantitative analysis of NLRP3 (E), caspase-1 (F), GSDMD (G), and IL-1β (H) expression, *n* = 5 per group. Positive cells were shown as red arrows. Relative expression levels of *Nlrp3* (I), caspase1 (J), *Gsdmd* (K), and IL1β (L) mRNA, *n* = 3 per group. Scale bar: 100 µm; all data presented as means ± standard deviations. *P < 0.05; ***P < 0.001; ^#^*P* < 0.05; ^##^*P* < 0.01; ^###^*P* < 0.001. OA: Osteoarthritis; IHC: Immunohistochemistry; NLRP3: NOD-like receptor protein 3; GSDMD: Gasdermin D; IL-1β: Interleukin-1β.

To test whether the degree of chondrocyte pyroptosis is related to the activation of autophagy, we performed IHC staining and RT-qPCR for NLRP3, caspase-1, GSDMD, and IL-1β on knee articular cartilage collected two and eight weeks after DMM surgery. The IHC results showed that NLRP3 (Figure 4A and 4E), caspase-1 (Figure 4B and 4F), GSDMD (Figure 4C and 4G), and IL-1β (Figure 4D and 4H) positive cells were significantly increased in the model group compared with the sham group; and abnormally expressed NLRP3, caspase-1, GSDMD, and IL-1β were significantly reduced in the RAPA treatment group compared with the model group. The results of RT-qPCR showed that the mRNA levels of *Nlrp3* (Figure 4I), caspase-1 (Figure 4J), *Gsdmd* (Figure 4K), and IL-1β (Figure 4L) in the model group were significantly increased compared with those in the sham group, and the mRNA levels of abnormally expressed *Nlrp3*, caspase-1, *Gsdmd*, and IL-1β were significantly reduced in the RAPA treatment group compared with those in the model group.

### Effect of autophagy on subchondral bone remodeling in OA

**Figure 5.**
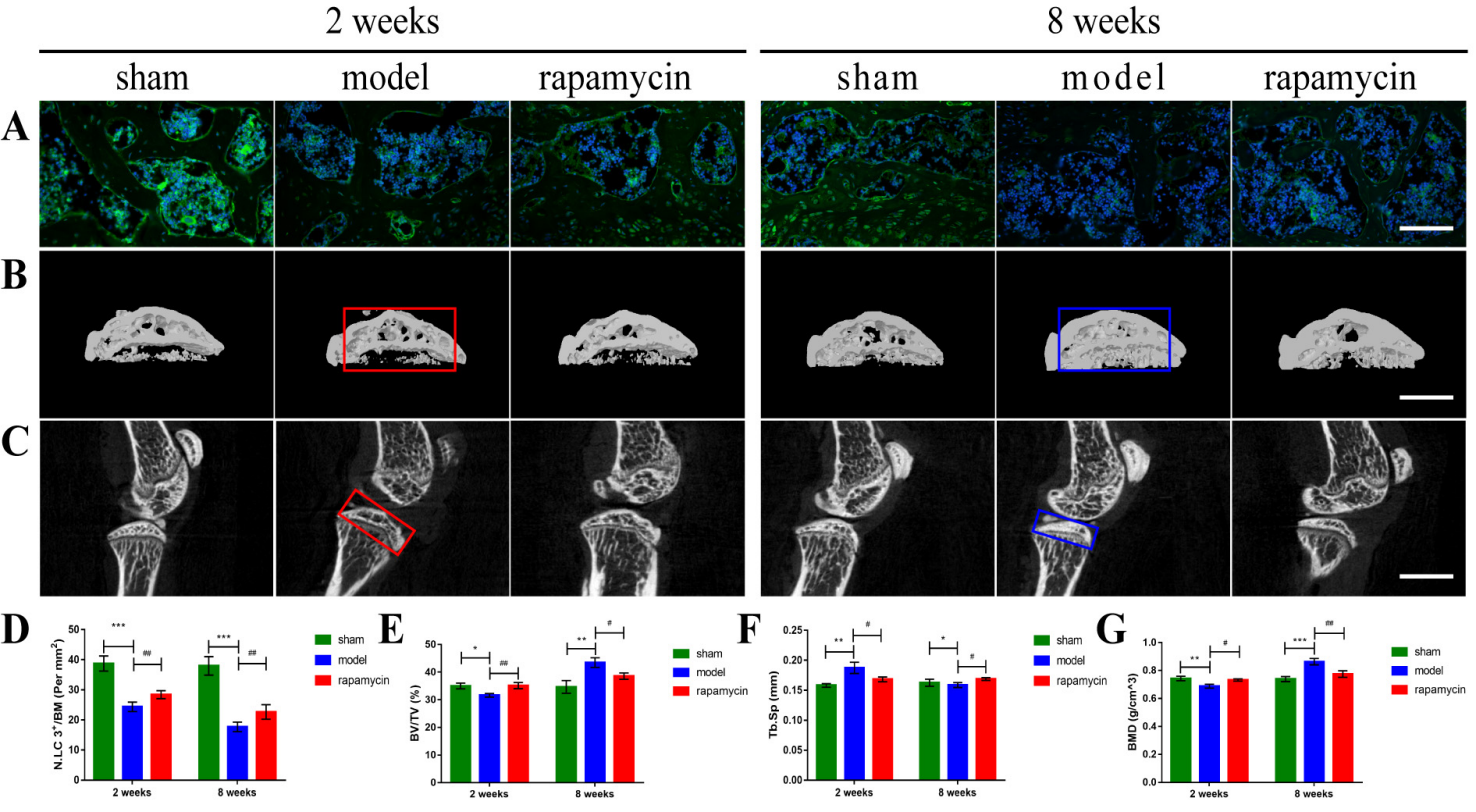
**Effect of autophagy on subchondral bone remodeling in a mouse OA model.** Representative pictures of IF staining (A) (scale bar: 100 µm), micro-CT two-dimensional reconstruction (B), and two-dimensional scanning (C) (scale bar: 2000 µm) of the subchondral bone of the knee joints at two and eight weeks after DMM surgery in mice, and quantitative analysis of LC3 expression (D), BV/TV (E), Tb.Sp (F) and BMD (G) in subchondral bone. Subchondral bone loss in the tibia is highlighted using a red box. Increases in bone mass and bone sclerosis are highlighted using a blue box, *n* = 3 per group, all data presented as means ± standard deviations. *P < 0.05; **P < 0.01; ***P < 0.001; ^#^*P* < 0.05; ^##^*P* < 0.01. OA: Osteoarthritis; IF: Immunofluorescence; micro-CT: Microcomputed tomography; DMM: Destabilization of the medial meniscus; LC3: Light chain 3; BV/TV: Bone volume fraction; Tb.Sp: Trabecular separation; BMD: Bone mineral density.

To assess the effect of autophagy on subchondral bone remodeling in OA, we performed IF staining and micro-CT scanning on the tibial subchondral bone of mouse knees collected two and eight weeks after DMM surgery. The results of IF showed that the expression of LC3 in the subchondral bone of the model group was significantly decreased compared with that in the sham group, and the abnormally expressed LC3 was significantly increased in the RAPA treatment group compared with the model group (Figure 5A and 5D). The results of micro-CT showed that in the early stage of OA (two weeks), compared with the sham group, the model group showed pathological changes of osteopenia in the tibial subchondral bone, decreased BV/TV and BMD, and increased Tb.Sp. Compared with the model group, treatment with RAPA alleviated the trend. In the late stage of OA (eight weeks), the model group showed pathological changes of increased bone mass in the tibial subchondral bone, increased BV/TV and BMD, and decreased Tb.Sp compared with the sham group. Compared with the model group, RAPA treatment alleviated these trends (Figure 5B, 5C, and 5E– 5G).

### Effect of autophagy on the number of osteoclasts in OA subchondral bone

**Figure 6.**
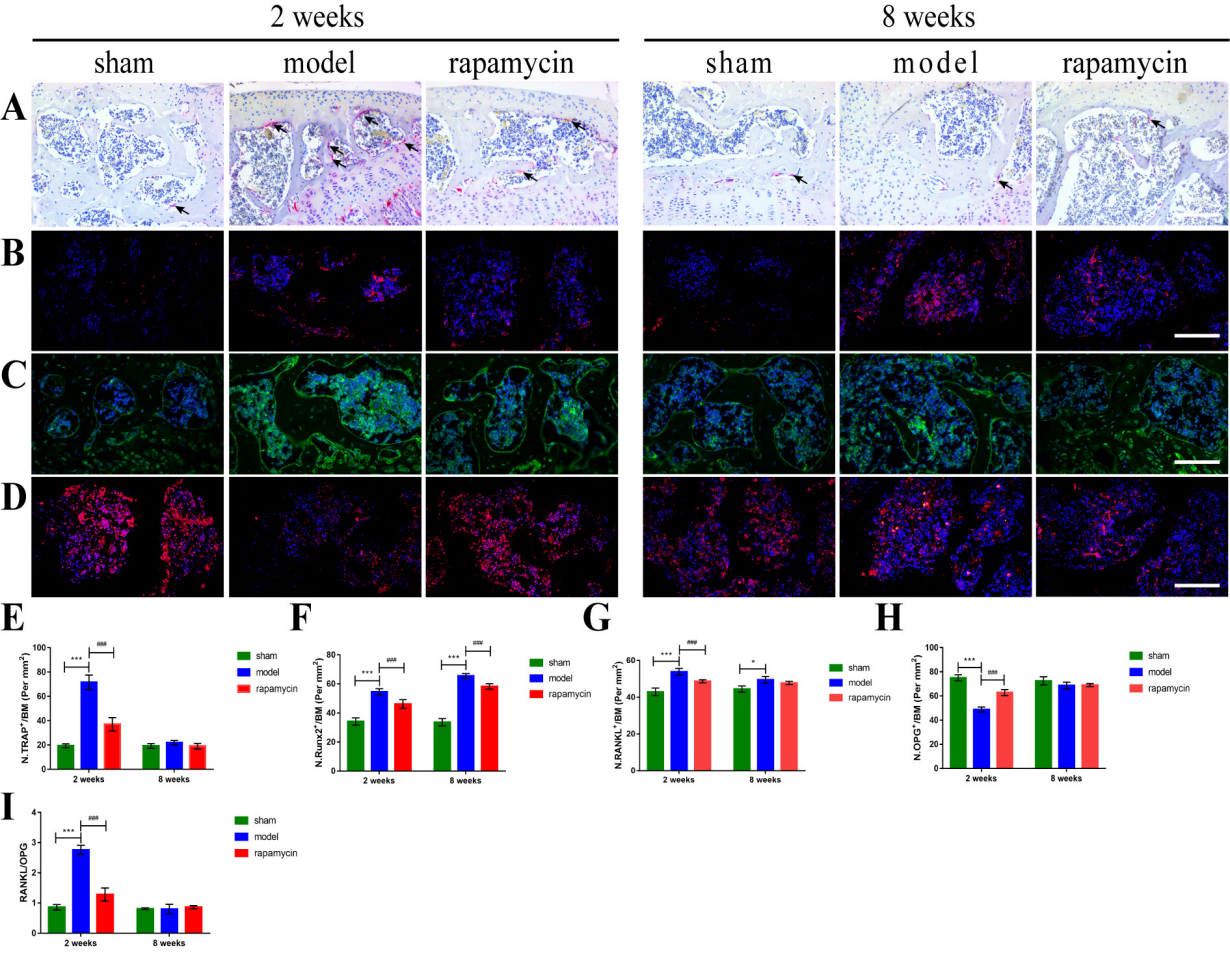
**Effect of autophagy on osteoclasts and osteoblasts in subchondral bone of OA mice.** TRAP staining (A) (scale bar: 200 µm) and IF staining of Runx2 (B), RANKL (C) and OPG (D) (scale bar: 100 µm) in knee subchondral bone at two and eight weeks after DMM surgery in mice. The number of mature osteoclasts (E), the expression of Runx2 (F), RANKL (G) and OPG (H), and the ratio of RANKL to OPG (I) in subchondral bone were quantitatively analyzed. Mature osteoclasts were shown as black arrows, *n* = 5 per group, all data presented as means ± standard deviations. *P < 0.05; ***P < 0.001; ^###^*P* < 0.001. OA: Osteoarthritis; DMM: Destabilization of the medial meniscus; TRAP: Tartrate-resistant acid phosphatase; Runx2: Runt-related transcription factor 2; RANKL: Receptor activator for nuclear factor-κB ligand; OPG: Osteoprotegerin; IF: Immunofluorescence.

To assess the effect of autophagy on osteoclasts number in OA subchondral bone, we performed TRAP staining and IF scanning on the tibial subchondral bone of mouse knees collected two and eight weeks after DMM surgery. The results of TRAP staining showed that in the early stages of OA (two weeks), the number of osteoclasts in the tibial subchondral bone was significantly increased in the model group compared with the sham group, and abnormally increased osteoclasts were significantly reduced in the RAPA group compared with the model group. The number of osteoclasts decreased in the late-OA (eight weeks) group compared with the early-OA group (two weeks), and there was no statistically significant difference between the sham, DMM, and RAPA groups (Figure 6A and 6E). The results of IF showed that in the early stages of OA (two weeks), the expression of receptor activator for NF-κB ligand (RANKL) in the subchondral bone of the model group was significantly increased compared with the sham group, and abnormally expressed RANKL was significantly reduced in the RAPA group compared with the model group (Figure 6C and 6G). The expression of osteoprotegerin (OPG) in the subchondral bone of the model group was significantly decreased compared with that of the sham group, and abnormally expressed OPG was significantly increased in the RAPA group compared with the model group (Figure 6D and 6H). Meanwhile, the ratio of RANKL to OPG in the subchondral bone of the model group was significantly increased compared with the sham group, and the abnormally ratio of RANKL to OPG was significantly reduced in the RAPA group compared with the model group (Figure 6I).

### Effect of autophagy on osteoblastogenesis in OA subchondral bone

To assess the effect of autophagy on osteoblastogenesis in OA subchondral bone, we performed IF staining of tibial subchondral bone from mouse knees collected two and eight weeks after DMM surgery. The results of IF showed that the expression of runt-related transcription factor 2 (Runx2) in the subchondral bone was significantly increased in the model group compared with the sham group, and the abnormally expressed Runx2 was significantly reduced in the RAPA treatment group compared with the model group (Figure 6B and 6F).

## Discussion

Accumulating evidence suggests that cartilage degeneration resulting from chondrocyte pyroptosis accelerates the pathological progression of OA [29, 30]. Meanwhile, abnormal bone remodeling of the subchondral bone disrupts the microarchitecture of the subchondral bone and accelerates cartilage degeneration [31]. It has been revealed that the activation of autophagy can reduce the occurrence of pyroptosis and delay the progression of the disease in chronic inflammatory diseases [32]. Activation of autophagy can mediate osteoclast differentiation [33] and osteoblast mineralization [34] in the subchondral bone of the knee. However, the question arises whether autophagy delays the pathological progression of OA by inhibiting the occurrence of chondrocyte pyroptosis as well as reversing subchondral bone remodeling. In this study, OA was induced in C57BL/6 mice using DMM, and the effect of autophagy on pyroptosis of OA chondrocytes and subchondral bone remodeling was investigated.

Pyroptosis is closely associated with a variety of chronic diseases [35]. As a key step in the initiation of pyroptosis, the NLRP3 inflammasome is the most extensively studied inflammasome, and its aberrant activation is associated with a variety of acute types of chronic inflammation [36–38]. Although NLRP3 has a potential role in a variety of chronic inflammatory diseases, we focused on OA. A recent study suggested that the NLRP3 inflammasome has the potential to be a therapeutic target for novel anti-OA drugs [39]. So far, the specific mechanism by which NLRP3 inflammasome activation regulates the development of pyroptosis in OA chondrocytes is still unknown. Uric acid in the synovium of OA patients has been demonstrated to enhance the activation of pre-IL-18 and pre-IL-1β by activating NLRP3 inflammasome, and high levels of inflammatory factors in the synovium in turn aggravate the pathological progression of OA [40]. The NLRP3/IL-18/IL-1β axis was abnormally activated in the synovial fluid of mice with collagen-induced arthritis, while the expression of NLRP3 positively correlated with the severity of knee OA [41]. Calcium phosphate crystals and hydroxyapatite crystals are found in the joint of OA and are well-known activators of NLRP3 inflammasome, which can trigger the activation of NLRP3 inflammasome by K^+^ efflux, ROS, and lysosomal destabilization [42–44]. It has also been shown that obese people have higher MMP activity compared with normal-weight people, which is accompanied by more inflammasome activation and can eventually lead to chondrocyte pyroptosis [39]. This evidence suggests an important role of NLRP3 inflammasome in the development of OA. Our study showed that the expression levels of NLRP3 in chondrocytes were significantly increased compared with the sham group in both early (two weeks) and late (eight weeks) stages of OA. Correspondingly, the NLRP3/caspase-1/GSDMD/IL-1β axis, led by NLRP3, was also abnormally activated compared to the control group. This suggests that abnormal activation of the NLRP3 inflammasome in OA chondrocytes mediated chondrocyte pyroptosis and may partially affect the pathological progression of OA. Interestingly, we also found that autophagy inhibits the occurrence of chondrocyte pyroptosis after activation of autophagy by RAPA. We have reason to speculate that inhibition of chondrocyte pyroptosis by autophagy may be a potential strategy to alleviate cartilage degeneration in knee OA. As an important part of the occurrence of pyroptosis, the treatment of NLRP3 inflammasome targeting autophagy activation may be a new treatment for OA [39].

**Figure 7.**
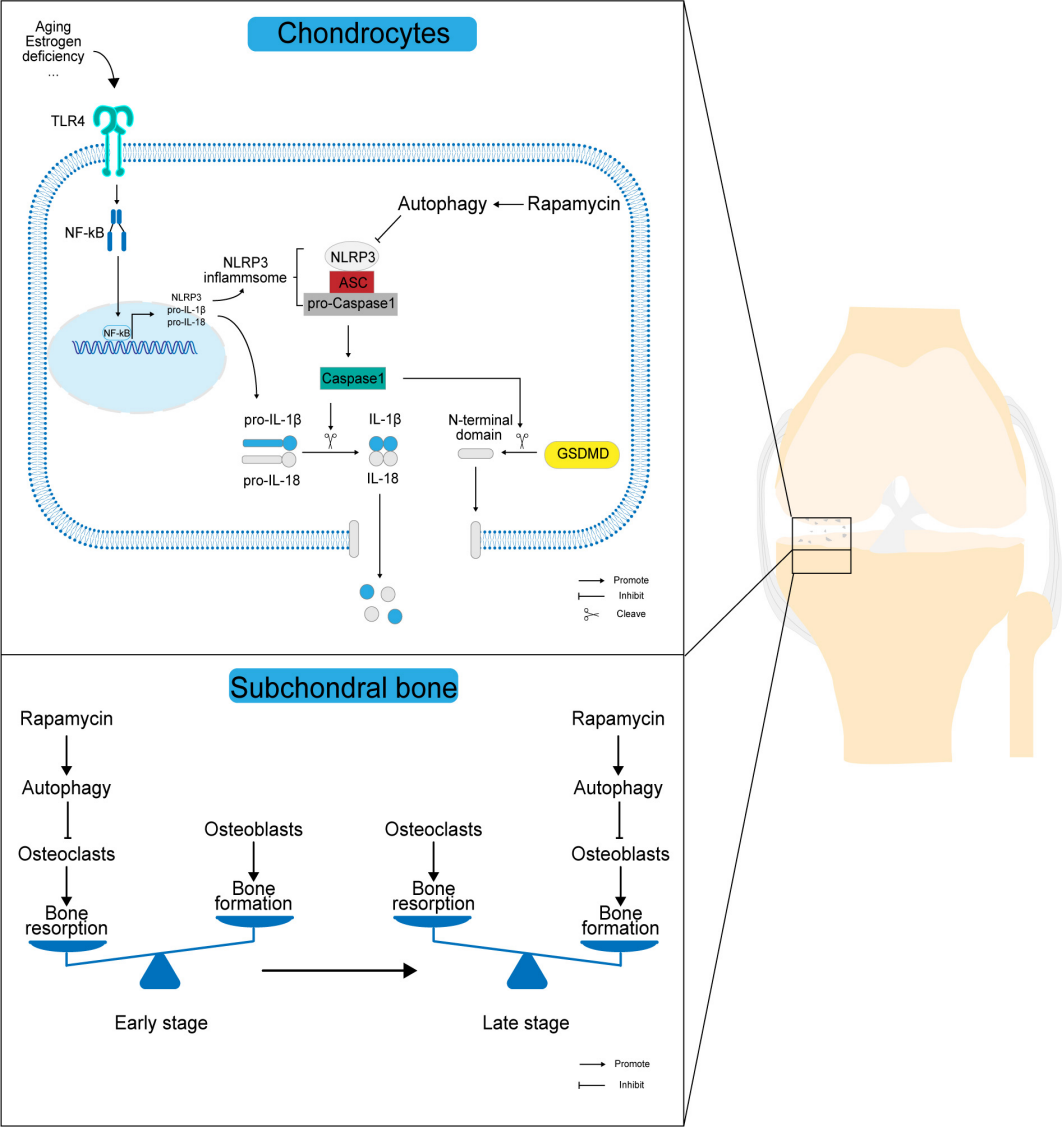
**Scheme of the mechanism by which autophagy affects the progression of OA pathology.** In OA chondrocytes, rapamycin activates autophagy while upregulating LC3 expression in chondrocytes, and highly expressed LC3 downregulates NLRP3 inflammasome activation while inhibiting the extracellular release of IL-1β and IL-18 through the NLRP3/caspase-1/GSDMD axis, thus reducing the destruction of articular chondrocyte membranes and the release of inflammatory mediators in the joint cavity. In OA subchondral bone, the activation of autophagy mainly maintains the dynamic balance of subchondral bone resorption and bone formation by inhibiting osteoclast-mediated bone resorption in the early stages and osteoblast-mediated bone formation in the late stages. OA: Osteoarthritis; LC3: Light chain 3; IL: Interleukin; NLRP3: NOD-like receptor protein 3; GSDMD: Gasdermin D; IL-1β: Interleukin-1β; TLR4: Toll-like receptor 4; NF-κB: Nuclear factor kappa B; ASC: Apoptosis-associated specific protein containing a C-termin caspase recruitment domain.

Autophagy is a cellular homeostatic mechanism that degrades and restores dysfunctional proteins and organelles to maintain homeostasis of the intracellular environment [45]. It has been demonstrated that autophagy can protect the integrity of cartilage [46]. This suggests that autophagy plays an important role in cartilage protection in OA. In a mouse OA model, intra-articular injection of RAPA decreased mammalian target of rapamycin (mTOR) expression in articular cartilage and delayed its degradation [13]. This suggests that activation of autophagy in chondrocytes delays OA progression by regulating cells metabolism and pyroptosis. It has been found that inhibition of autophagy can lead to abnormal cell function and cell death [47]. Autophagy downregulates NLRP3 inflammasome expression and prevents its overactivation by inhibiting the production of inflammatory mediators and mitochondrial degradation [48]. Meanwhile, LC3 is a structural and functional molecule formed by autophagy, and its expression level reflects the level of autophagy activation [49, 50]; LC3 is significantly downregulated in OA [51]. This is consistent with our study in which we evaluated the regulation of NLRP3 inflammasome in OA mouse chondrocytes by autophagy using intraperitoneal injection of RAPA. We found that RAPA upregulated LC3 and decreased NLRP3 expression in articular cartilage, while inhibiting the expression of the catabolic factor MMP-13, promoting the expression of the anabolic factor Col II, reducing the pathological score of OA, and restoring HC and CC thickness. Normally, the synthesis, repair, and degradation of articular cartilage matrix can maintain a state of balance, and this balance of cartilage matrix is broken when the knee joint is affected by various factors, such as mechanical and biochemical factors [52]. The MMPs are a large group of zinc-dependent endoproteases with similar structures that constitute the most important proteolytic system for extracellular matrix degradation and play an important role in the conversion of OA cartilage and matrix [53]. Studies have shown that MMP-13 is mainly secreted by chondrocytes and synoviocytes, and can cause extracellular matrix degradation by downregulating the expression of Col II, leading to cartilage degeneration [54]. Our study confirmed that in OA chondrocytes, autophagy can inhibit the activation of the NLRP3 inflammasome, which in turn delays the pathological progression of OA. The activation of the NLRP3 inflammasome is a key link in the development of pyroptosis, which provides strong support for autophagy to delay the pathological progression of OA by inhibiting chondrocyte pyroptosis mediated by NLRP3 inflammasome.

The subchondral bone plays an important role in constituting and maintaining the normal structure and function of the knee joint [55]. An animal experiment has shown that early injury of subchondral bone may precede cartilage degeneration, mainly manifested as osteopenia due to enhanced bone resorption, followed by subchondral osteosclerosis due to enhanced bone formation [56]. Currently, reversal of subchondral bone remodeling can serve as a potential target to delay the progression of OA pathology [57]. The OPG/RANKL/RANK system plays an important role in OA subchondral bone remodeling [58]. The OPG/RANKL ratio can be used as an important indicator of the level of osteoclast activity and regulate bone metabolism [59]. Li et al. [60] found that OPG levels were decreased, and RANKL levels were increased in subchondral bone of early OA *in vivo*. According to our previous study, the RANK/RANKL/OPG axis played a key role in the process of osteoclast activation in the subchondral bone of early OA [61, 62]. In this study, we found that BV/TV and BMD in the subchondral bone at the early stage of OA (two weeks) decreased, Tb.Sp, RANKL/OPG, and osteoclast formation increased, and bone resorption was correspondingly enhanced. We found that activation of autophagy by RAPA could reverse this trend. However, in the late stages of OA (eight weeks), the number of osteoclasts in the subchondral bone was significantly lower than that in the early stages of OA (two weeks), bone resorption was significantly reduced, and bone formation played a major role during this period.

As the main transcription factor of bone formation, Runx2 plays an important role in the differentiation of intercellular progenitor cells into osteoblasts [63]. This is in agreement with our study, where BV/TV and BMD increased in the subchondral bone when OA progressed from the early (two weeks) to the late (eight weeks) stages. The Tb.sp decreased and elevated Runx2 expression indicated increased osteoblast formation and correspondingly enhanced bone formation, whereas RAPA reversed the trend by activating autophagy. Therefore, the inhibition of bone formation in the subchondral bone in the late stage of OA may be caused in part by decreased Runx2 due to autophagy activation. In addition, we also found elevated expression of Runx2 in the subchondral bone at the early stage of OA (two weeks), representing enhanced osteogenesis, but the effect of bone resorption due to massive osteoclastogenesis at this stage was stronger than that of bone formation. These results indicate that the pathological phenomenon of abnormal bone remodeling occurred in OA subchondral bone, with bone resorption predominating in the early stages and bone formation predominating in the late stages. At the same time, treatment with RAPA can improve the microarchitecture of the subchondral bone, improve abnormal bone remodeling of the subchondral bone, and mainly inhibit bone resorption in the early stages, while predominately inhibiting bone formation in the later stages. Based on the above results, it is reasonable to speculate that autophagy reverses abnormal bone resorption mainly by inhibiting osteoclastogenesis through the RANK/RANKL/OPG axis in the early stage of OA. It reversed abnormal bone formation mainly by inhibiting osteoblastogenesis in the late stages of OA.

Although this study provided some experimental data and a theoretical basis, it had some limitations. In particular, it was limited to an animal model—the role of autophagy in the development of OA by inhibiting chondrocyte pyroptosis and reversing abnormal bone remodeling in the subchondral bone needs to be explored further at the *in vitro* level. Given that systemic administration of RAPA may have the side effects of thrombocytopenia [64] and delayed cell proliferation [65], the model of administration we chose may have some impact on the results. We believe that an intra-articular injection will be a great option in future.

## Conclusion

We demonstrated a novel role for autophagy in the progression of OA pathology in a mouse model. We have shown that activation of autophagy by RAPA delays the pathological progression of OA by inhibiting chondrocyte pyroptosis and reversing abnormal bone remodeling in the subchondral bone (Figure 7). With regard to the positive role of autophagy activation by RAPA in the OA development, further studies with large samples of clinical data are needed.

## Acknowledgments

We would like to thank AME Editing Service (https://editing.amegroups.com/) for English language editing. The present study was supported by the Scientific Research Project from Ningxia Province (No. 2020AAC03390), the Scientific Research Project from Ningxia Province (No. 2020AAC03392), and the National Natural Science Foundation of China (No. 8216090355).

**Conflicts of interest:** Authors declare no conflicts of interest.

**Funding:** Authors received no specific funding for this work.
